# Supplementary material for: Flavonoid Metabolism in Tetrastigma hemsleyanum Diels et Gilg Based on Metabolome Analysis and Transcriptome Sequencing
Source: Molecules. 2022 Dec 22;28(1):83. doi: 10.3390/molecules28010083 (PMC9821845; doi:10.3390/molecules28010083)
Supplement: Supplementary file 1 [file molecules-28-00083-s001.zip › molecules-1991097-supplementary.pdf]

Table S1 Concentration of flavonoid Active ingredients in different parts of *T. hemsleyanum*

Active ingredients	Leaf (ug/g)	Root tuber (ug/g)
Isorhamnetin-3G	0.046±0.001^{ns}	0.045±0.001
Isorhamnetin	0.030±0.007^{ns}	0.032±0.002
Kaempferol	—	0.0244±0.005***
Eriodictyol	0.012±0.003*	0.002
Myricetin	0.012**	0.001
Baicalin	0.007±0.002***	—
Chrysoeriol	0.006±0.001*	0.0002
Diosmetin	0.005***	0.0002
Naringenin	0.004±0.001^{ns}	0.006
Apigenin	0.002^{ns}	0.002
Quercitrin	0.002 ^{ns}	0.002
Apigenin-7-G	0.002***	—

Different symbol means significant difference at 0.05 level.
